# Examining Injury Severity of Pedestrians in Vehicle–Pedestrian Crashes at Mid-Blocks Using Path Analysis

**DOI:** 10.3390/ijerph17176170

**Published:** 2020-08-25

**Authors:** Haorong Peng, Xiaoxiang Ma, Feng Chen

**Affiliations:** The Key Laboratory of Road and Traffic Engineering, Ministry of Education, Tongji University, 4800 Cao’an Road, Jiading, Shanghai 201804, China; 1810095@tongji.edu.cn (H.P.); xiaoxiang.ma@tongji.edu.cn (X.M.)

**Keywords:** pedestrian, injury severity, mid-blocks, path analysis

## Abstract

Walking is a sustainable mode of transport which has well established health and environmental benefits. Unfortunately, hundreds of thousands of pedestrians lose their lives each year over the world due to involvement in road traffic crashes, and mid-blocks witness a significant portion of pedestrian fatalities. This study examined the direct and indirect effects of various contributing factors on the pedestrian injury severity in vehicle–pedestrian crashes at mid-blocks. Data of vehicle–pedestrian crashes during 2002–2009 were extracted from the NASS-GES, with pre-crash behaviors and injury severity included. The SEM path analysis method was applied to uncover the inter-relationships between the pedestrian injury severity and various explanatory variables. Both the direct and indirect effects of these explanatory variables on the pedestrian injury severity were calculated based on the marginal effects in the multinomial and ordered logit models. The results indicate some variables including number of road lanes and the age of pedestrian have indirect impacts on the injury severity through influencing the pre-crash behaviors. Although most indirect effects are relatively small compared with the direct effects, the results in this study still provide some valuable information to improve the overall understanding of pedestrian injury severity at mid-blocks.

## 1. Introduction

Walking is a sustainable mode of transport which benefits public health and contributes to reducing global warming [1,2]. To reduce gas emissions and improve public health, walking trips are encouraged by many governments around the world in recent years [3]. Undoubtedly, the safety issue for pedestrians is the most critical concern which needs to be addressed when promoting walking. However, as the most vulnerable road users, pedestrians are exposed to a higher risk of injury and fatality in traffic crashes, which results in hundreds of thousands of pedestrian fatalities each year over the world [3]. In the United States, pedestrian deaths increased by 53% from 2009 to 2018. Specifically, 6283 pedestrians were killed in traffic crashes in 2018, accounting for 17% of all traffic fatalities [4]. Although great efforts have been made to improve pedestrian safety, the upward trend in pedestrian fatalities revealed by the traffic safety data is concerning [5]. Therefore, more studies on the pedestrian safety are needed to uncover the key factors contributing to pedestrian injuries and fatalities in traffic crashes, as well as the relationships between the pedestrian injury severity and various risk factors.

Traffic crashes involving pedestrians occur at both intersection and mid-block locations [6]. Mid-block crossings witnessed a significant portion of pedestrian fatalities; for example, in Melbourne metropolitan area, 49% of pedestrian fatalities occurred at mid-block crossings during 2010–2016 [7]. Besides, due to the higher vehicle speed, vehicle–pedestrian crashes occurred at mid-blocks are more likely to cause severe injuries and fatalities compared with traffic crashes involving pedestrians at intersections [8]. While many previous studies have been conducted to investigate the factors contributing to the severity of vehicle–pedestrian crashes, most of them focused on traffic crashes at intersections. Only a few of studies focused on vehicle–pedestrian crashes at mid-blocks. Thus, it is essential to explore the contributing factors of vehicle–pedestrian crash at mid-block crossings.

The pre-crash behavior of the pedestrian, a key contributing factor in traffic crashes, has a significant influence on the resulting injury severity in the vehicle–pedestrian crash at the mid-block [9]. To examine the impact of the pedestrian’s pre-crash behavior on the injury severity at mid-block crossings, some statistical models, such as the multinomial logit model and the ordered probability model, have been established to quantify the effect. In these studies, the influence of various potential factors (including time characteristics, crash features, environmental conditions, pedestrian characteristics, and roadway attributes) on the injury severity levels were also explored and presented. By quantifying direct effects of contributing factors on the injury severity levels, these above-mentioned research efforts made valuable contributions to the overall understanding of vehicle–pedestrian crashes at mid-blocks. However, besides direct effects, some associated factors might have indirect effects on the injury severity levels since the relationships between injury severity and covariates are more nuanced and the variables are possibly inter-related. For example, the pedestrian characteristic may have indirect effects on the injury severity through influencing the pre-crash behavior. Those inter-relationships could be untangled by using the method known as path analysis, a statistical analysis method for Structural Equation Modeling (SEM), which has been applied for many studies concerning driving behaviors and traffic crashes [10,11,12,13].

To the authors’ knowledge, few, if any, published studies have examined both the direct and indirect effects of contributing factors on the injury severity in vehicle–pedestrian crashes at mid-blocks. Hence, there is a gap in the literature. The objective of this study was to investigate relationships between injury severity outcomes and various contributing factors using path analysis. The multinomial logit model was used to estimate the effects of some variables including pedestrian characteristics and roadway attributes on the pre-crash behaviors of pedestrians. The ordered logit model was applied to estimate the direct associations between the pedestrian injury severity and its explanatory variables. Based on the marginal effects in these two models, both direct and indirect effects of explanatory variables on the injury severity were examined. The results presented in this study are expected to facilitate the overall understanding of pedestrian injury severity at mid-blocks and make a contribution to the improvement of pedestrian safety.

The rest of this paper is organized as follows. A review of previous studies is provided in Section 2. The description of the data used for analysis is presented in Section 3. Section 4 introduces the path analysis, as well as the multinomial and the ordered logit models. The results and discussions are given in Section 5, followed by some concluding remarks in Section 6.

## 2. Literature Review

This study examined both the direct and indirect effects of various factors on the injury severity of pedestrians in traffic crashes at mid-block crossings. Accordingly, the literature review focuses on two main topics, namely analysis of vehicle–pedestrian crashes at mid-blocks and application of SEM (especially the path analysis method) on traffic crashes. 

In recent years, much more attention has been paid to analyzing the pedestrian crashes at mid-blocks. The risk of pedestrian crashes at mid-block crossings was evaluated by developing a Poisson regression model, indicating that the pedestrian crash risk is significantly influenced by a combination of interactive risk factors including the road features and the traffic volume [14]. Focusing on child pedestrians, conditional logistic regression was used to contrast collision risk at mid-block and intersection locations, suggesting that some factors associated with the collision risk differ between the two location types [6]. Using multilevel mixed effects Poisson models, Quistberg et al. [15] estimated the risk of pedestrian collisions at intersections and mid-blocks in Seattle with the lack of behavioral factors for both drivers and pedestrians, which resulted in their contributions to pedestrian collisions being unable to be analyzed. Chen et al. [16] also suggested that behaviors of different road users at mid-blocks should be taken into account when analyzing pedestrian fatality risk in accidents. As for pedestrian crash severity at mid-blocks, Pour et al. [5] applied boosting decision trees to identify the contributing factors of the injury severity, showing that neighborhood social characteristics influenced the severity of pedestrian crashes significantly. Kwayu et al. [8] discerned human, environmental and roadway factors associated with pedestrian–vehicle crashes at undesignated mid-block areas in Michigan State, and the results show that the most influential predictors of pedestrian fatalities were the lighting conditions, pedestrian age, and traffic volume. In these aforementioned studies, the influence of the pedestrian behavior on the crash risk or the injury severity was not examined due to the lack of the corresponding data. By extracting the traffic crash records involving the pre-crash behavior of non-motorists from the National Automotive Sampling System—General Estimates System (NASS-GES), Dong et al. [9] examined the risk factors influencing the injury severities of non-motorists (including pedestrians and cyclists) at mid-block crossing, and the role of the non-motorists’ pre-crash behavior in their resulting injury severity outcomes was also explored based on a mixed logit model. Besides, some typical studies [17,18,19] relating to the traffic crash severity also provided useful references to the study of pedestrian injury severity.

Although several studies have examined the influence of various factors including the pedestrians’ pre-crash behavior on the pedestrian injury severity at mid-blocks [9], the indirect effects of some associated factors have not been explored yet. Since factors such as age, gender, and pedestrian volume may affect the behavior of a pedestrian crossing the road [20], these factors would have indirect effects on the injury severity of the pedestrians in traffic crashes at mid-block crossings. However, this kind of inter-relationships cannot be discovered by a single regression model, such as the multinomial logit model, ordered logit/probit model, and the mixed logit model [21]. SEM is required to examine both the direct and indirect effects of contributing factors on the outcome variable, which has been employed in many studies relating to traffic crashes [10,11,12,13]. Using SEM with different model structures, Wang and Qin [12] explored the direct and indirect contribution of various factors to the severity of single-vehicle crashes. Liu et al. [11] quantified the direct and indirect associations of passive and active controls with pre-crash behaviors and crash outcomes in terms of injury severity by using path analysis, in which two models were estimated, one for pre-crash driving behaviors, and another model for injury severity. Lee et al. [10] analyzed the rainfall and traffic accident data from 2007 to 2015 using SEM with the aim to identify the relationship between the accident severity and rain-related factors. Shaaban et al. [13] employed SEM to examine the indirect relationship between injury severity of red-light-running-related crashes and some contributing factors. These previous studies provided good examples for the application of SEM in the present study.

## 3. Data Description

The data for this study were obtained from (NASS-GES. The NASS-GES dataset contains representative crash samples selected from police-reported crashes by the data collectors in 60 geographic sites across the US [22]. Since a key variable about pedestrian behavior (pre-crash behavior) was discontinued in 2010, eight years of records (2002–2009) were chosen as the original dataset. Two primary selection criteria were applied to select records from the original dataset: the location of the vehicle–pedestrian crash is the mid-block and the pre-crash behavior of the pedestrian is recorded completely and clearly. The mid-blocks witnessed about 30% of the vehicle–pedestrian crashes in the original dataset, thus the location criterion excluded nearly 70% of the records. Most of the remaining records did not contain complete and clear description of the pre-crash behavior of the pedestrian. Since the pre-crash behavior is a critical factor in this study with the aim of investigating the indirect effect of some variables on the pedestrian injury severity through the pre-crash behavior, imputation methods based on other variables are not reliable enough to make the missing pre-crash behavior complete. Therefore, only records with the complete and clear description of pedestrian pre-crash behaviors were selected to form a final dataset used in this study. The final dataset contains 3653 records, which accounts for a relatively small proportion of the NASS-GES dataset, but its size is large enough to trigger the modeling of the path analysis applied in this study. Five categories of information were involved in the final dataset, including pedestrian characteristics, vehicle characteristics, roadway features, environmental conditions, and crash attributes.

The summary description of the severity outcome and potential explanatory factors is presented in Table 1. In the original data from NASS-GES, the pedestrian injury severity was classified into five levels. Since only 10 records in the final dataset were labeled as No Injury, which account for only 0.27% of the total records, the No Injury level and the Possible Injury level were combined into one category. Therefore, four levels of injury severity are presented in Table 1, namely: No Injury/Possible Injury (NIPI, 157 records), Non-Incapacitating Evident Injury (NIEI, 1993 records), Incapacitating Injury (ICI, 1238 records), and Fatal Injury (FI, 265 records).

In Table 1, the meanings of the variables relating to pedestrian characteristics, vehicle characteristics and roadway features are easy to understand through the variable name. As for the environmental conditions, the variable Time of Day was processed into three subcategories: Nighttime (8 p.m. to 7 p.m.), Peak time (7 a.m. to 10 a.m. or 5 p.m. to 8 p.m.), and Other time (not listed in the table). The surface condition indicated the dry or wet state of the roadway surface when the vehicle–pedestrian crash occurred, which was classified under the environmental conditions since it was mainly determined by the weather instead of the roadway features. Concerning the vehicle–pedestrian crash attributes, the first point of impact was chosen as one of the potential explanatory factors, which was classified into three subcategories (the front, the right side, and the left side of the vehicle). Besides, the pedestrian behavior before crash, also named the pre-crash behavior, was classified into five subcategories in this study based on the coding scheme of the GES coding and editing manual [23]. The detailed descriptions of these pre-crash behavior subcategories are as follows: (1) Darting or Running Into Road means the pedestrian’s pre-crash behavior of crossing the road can best be described as an impulsive or sudden darting, running, jogging, etc.; (2) Improper Crossing indicates that the person was crossing a road by walking or crawling before the crash and was not in the continuation of jogging/running or did not engage in a sudden or impulsive dart, run, etc.; (3) Activity in Roadway includes playing in the road before the vehicle arrived and working in the road because of his/her job; (4) Inattentive represents that the pedestrian was standing, sitting or lying, and perhaps waiting inattentively; and (5) Other Action means that the pedestrian did take an action, which cannot best fit in the subcategories specifically described above.

## 4. Method

The objective of this study was to examine both the direct and indirect effects of contributing factors on the injury severity in vehicle–pedestrian crashes at mid-blocks. To this end, path analysis method of SEM was applied to establish the model and analyze these effects. Specifically, some factors can have a direct association with the injury severity and indirect effect through influencing the pre-crash behavior of the pedestrian in the traffic crash at mid-blocks.

**Figure 1:**
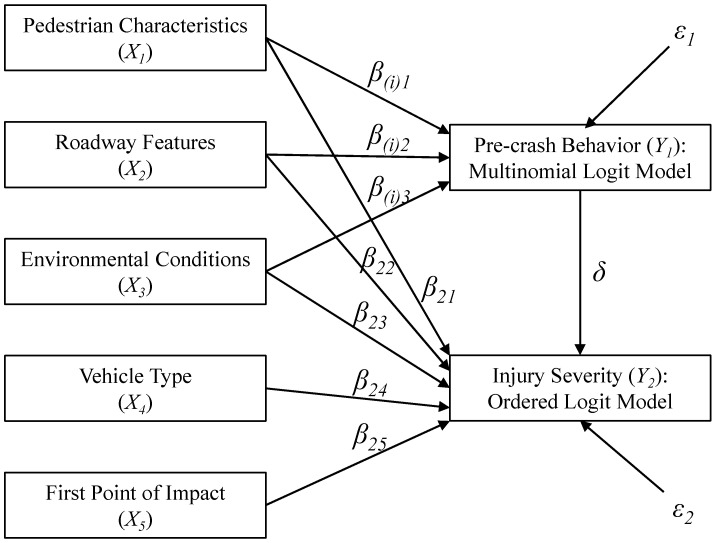
Conceptual framework for estimating effects of various factors on the injury severity.

The conceptual framework of the path analysis used in this study is shown in Figure 1. It should be noted that *X*_1_, *X*_2_, and *X*_3_ in Figure 1 represent several factors belonging to corresponding categories in Table 1 in order to make the path diagram more concise and explicit. For example, *X*_1_ represents Age and Gender factors of the pedestrian characteristics.

The path analysis structure in this study contains two models. The first one is a multinomial logit model, which is used to examine the effects of some variables on the pre-crash behavior of the pedestrian (*Y*_1_). The second model is used to uncover the associations between the injury severity (*Y*_2_) and contributing factors including the pre-crash behavior of pedestrian. Considering the ordinal nature of the injury severity, the ordered logit model was employed for its simplicity and ease of interpretation [11].

The following equations describe the path analysis structure:

(1) $$Y_{1}=X_{13}\beta_{\left(i\right)}+\varepsilon_{1}$$

(2) $$Y_{2}=X_{15}\beta_{2}+Y_{1}\delta +\varepsilon_{2}$$

where $Y_{1}$ is the pre-crash behavior of the pedestrian, including 5 subcategories; $X_{13}$ is a vector of explanatory variables in the multinomial logit model, which contains $X_{1}$, $X_{2}$, and $X_{3}$; $\beta_{\left(i\right)}$ is a set of coefficients corresponding to the *i*th behavior in the multinomial logit model (*i* = 1, 2, 3); $Y_{2}$ is the pedestrian injury severity measured on ordinal scale; $X_{15}$ is a vector of explanatory variables in the ordered logit model, including $X_{1}$, $X_{2}$, $X_{3}$, $X_{4}$, and $X_{5}$; $\beta_{2}$ is a set of coefficients of $X_{15}$ in the ordered logit model; δ is the association of the pre-crash behavior of the pedestrian with the injury severity; and $\varepsilon_{1}$ and $\varepsilon_{2}$ and error terms, which are assumed to be uncorrelated.

In the multinomial logit model, the conditional probabilities of each outcome category (i.e., one type of pre-crash behavior) is given by:

(3) $$\begin{array}{l} \mathrm{Pr}\left(Y_{1}=i\right)\\ =\;\frac{\exp\left(X_{13}\beta_{\left(i\right)}\right)}{\exp\left(X_{13}\beta_{\left(1\right)}\right)+\exp\left(X_{13}\beta_{\left(2\right)}\right)+\exp\left(X_{13}\beta_{\left(3\right)}\right)+\exp\left(X_{13}\beta_{\left(4\right)}\right)+\exp\left(X_{13}\beta_{\left(5\right)}\right)} \end{array}$$

In the second model, namely, the ordered logit model, the response variable is the pedestrian injury severity. In this study, $Y_{2}=1$ represents NIPI; $Y_{2}=2$ indicates NIEI; $Y_{2}=3$ represents ICI; and $Y_{2}=4$ is FI. The predicated probabilities of the pedestrian injury severity are:

(4) $$\mathrm{Pr}\left(Y_{2}=1\right)=\;\frac{\exp\left(\alpha_{1}-X\beta \right)}{1+\exp\left(\alpha_{1}-X\beta \right)}$$

(5) $$\mathrm{Pr}\left(Y_{2}=2\right)=\frac{\exp\left(\alpha_{2}-X\beta \right)}{1+\exp\left(\alpha_{2}-X\beta \right)}-\frac{\exp\left(\alpha_{1}-X\beta \right)}{1+\exp\left(\alpha_{1}-X\beta \right)}$$

(6) $$\mathrm{Pr}\left(Y_{2}=3\right)=\frac{\exp\left(\alpha_{3}-X\beta \right)}{1+\exp\left(\alpha_{3}-X\beta \right)}-\frac{\exp\left(\alpha_{2}-X\beta \right)}{1+\exp\left(\alpha_{2}-X\beta \right)}$$

(7) $$\mathrm{Pr}\left(Y_{2}=4\right)=1-\left[\mathrm{Pr}\left(Y_{2}=1\right)+\mathrm{Pr}\left(Y_{2}=2\right)+\mathrm{Pr}\left(Y_{2}=3\right)\right]=1-\frac{\exp\left(\alpha_{3}-X\beta \right)}{1+\exp\left(\alpha_{3}-X\beta \right)}$$

where *X* is a vector of explanatory variables including the pedestrian pre-crash behavior; *β* is a set of coefficients of *X*; and $\alpha_{1}$, $\alpha_{2}$, and $\alpha_{3}$ are the intercepts of linear form equations.

It should be noted that the equations in ordered logit model share one set of coefficients but with different intercepts, which is unlike the multinomial logit model where each outcome category possesses its own set of coefficients. Besides, to calculate the direct and indirect effects of explanatory variables on the injury severity, marginal effects for the multinomial and ordered logit models should be obtained. The marginal effect measures the change in the dependent variable associated with a unit change in an explanatory variable while keeping all other explanatory variables constant [24]. For more details about the marginal effects calculation by Stata, refer to StataCorp [25].

## 5. Results and Discussion

In this study, the software Stata [25] was applied to conduct the path analysis, which allowed two models to be estimated simultaneously. Table 2 and Table 3 present the results of the multinomial logit model for pre-crash behavior of the pedestrian and the ordered logit model for pedestrian injury severity, respectively. It should be noted that some statistically insignificant variables are also contained in the models, either because they are part of a type of subcategory or there is a need to include them with the aim of analyzing the inter-relationships. The maximum likelihood approach was used to estimate the coefficients for explanatory variables. Besides, to calculate the direct, indirect, and total effects of explanatory variables on the injury severity, the marginal effects are listed in Table 2 and Table 3.

In the multinomial logit model, one of the pre-crash behaviors—other action—was chosen to be the base level. The marginal effects indicate the probability change of four other types of pre-crash behaviors compared with the base outcome. As shown in Table 2, the age of pedestrian was found to be significantly associated with the pre-crash behaviors (Darting or running into road and Improper crossing) at mid-blocks. Compared with pedestrians at the base age (<25), the chances of darting or running into road are lower for the older pedestrians. The marginal effects show a much lower probability by 27.3%, 31.1%, and 27.1% of darting or running into road for pedestrians with the ages 25–45, 45–65, and >65, respectively. it seems reasonable because children and teenagers are more likely to be involved in darting or running when crossing the road at mid-blocks. In contrast, the older pedestrians are more likely to conduct the Improper crossing than the pedestrians at age <25, with 21.8% and 29.2% higher probabilities of improper crossing for the ages 45–65 and >65, respectively. Besides, the number of lanes also influences the probability of the Improper crossing significantly. The marginal effects show that, compared with the one- and two-lane roads, pedestrians have a higher chance to conduct the improper crossing at mid-blocks when crossing the three- (5.8% higher), four- (8.9% higher), or five-or-more-lane (7.3% higher) roads. In addition, environmental conditions exert an influence on the pre-crash behaviors of pedestrians. Nighttime decreases the probability of darting or running into road by 7.7% as compared to daytime but increases the chance of activity in road by 6.2%. Compared with the wet surface condition of the roadway, the dry surface results in a higher probability (7.1% higher) for pedestrians to be involved in darting or running into road.

Table 3 shows the model estimation results and the marginal effects of explanatory variables on injury severity for the ordered logit model. The model results suggest that four types of pre-crash behaviors were statistically significantly correlated to the pedestrian injury severity in vehicle–pedestrian crashes at mid-blocks. Compared with the reference category of the pre-crash behavior (Other action), the marginal effects indicate that all of these pedestrian pre-crash behaviors decreased the likelihood of ICI and FI while increasing the likelihood of NIPI and NIEI; For example, pedestrians who are inattentive before the vehicle–pedestrian crashes have a 14.3% higher probability of NIEI when crossing the road at mid-blocks. With respect to the vehicle type, heavier vehicles (light trucks, buses, and heavy trucks) tend to increase the likelihood of more severe pedestrian injuries and lighter vehicles (motorcycles) result in a lower probability of ICI (4.8% lower) and FI (2.1% lower) as compared to automobiles. This is reasonable and consistent with many previous studies associated with pedestrian injury severity [9,26,27]. The first point of impact also has a significant influence on the injury severity. As shown in Table 3, compared with the front point of the vehicle, both the left side and right side decrease the likelihood of ICI and FI in pedestrian–vehicle crashes at mid-blocks.

Since the vehicle type and the first point of impact are not included in the multinomial logit model, the effects of these two explanatory variables on the injury severity are presented in Table 3 completely. However, for other explanatory variables in both the multinomial and ordered logit models, the marginal effects in Table 3 only indicate their direct effects on the injury severity. Calculating the indirect effects of these explanatory variables on the injury severity requires combining the results of the multinomial and ordered logit models. The calculation processes are presented in Table 4, which takes the effects on the injury severity ICI for an example. For clarity, non-statistically significant effects were omitted. The multinomial logit model in Table 2 shows that the age over 65 is related to a 27.1% lower likelihood of darting or running into road, while this type of pre-crash behavior is associated with a 3.9% lower probability of ICI shown in Table 3. Thus, the indirect impact of the age >65 on increasing the likelihood of pedestrian suffering ICI is 27.1% × 3.9% = 1.1%. Accordingly, the indirect effect of the age >65 through other pre-crash behaviors on increasing or decreasing the ICI of pedestrians is 1.1%, −1.0%, 0.0%, and 0.0% respectively. Besides, the marginal effect in Table 3 indicates that pedestrians aged over 65 have a 15.2% higher likelihood to be involved in ICI as compared to pedestrians at the reference age group, which is a direct effect on the injury severity ICI. Therefore, the total effect of the age (>65) on the ICI is the sum of the direct and indirect effects, namely 15.3%. Similarly, the indirect and total effects of other explanatory variables on the ICI were obtained, as shown in Table 4.

In the same way, the indirect and total effects of all the explanatory variables in the multinomial logit model on the injury severity were calculated, as presented in Table 5. Although the indirect effects are relatively small compared with the direct effects for most of the explanatory variables, the path analysis method does provide valuable help to understand the inter-relationship between these explanatory variables and the injury severity. According to the total effects in Table 5, older people are more likely to suffer severe injuries in vehicle–pedestrian crashes, compared with young pedestrians (<25). Pedestrians aged over 65 have a 6.7% higher likelihood to death (i.e., FI) in vehicle–pedestrian crashes at mid-blocks, which is higher than the 4.1% for the age 45–65 and the 2.7% for the age 25–45. This result confirms that, as compared to the young, the older pedestrians are more physically vulnerable in traffic crashes, which has been documented in many previous studies [28,29].

Besides, the total effects in Table 5 indicate that the likelihood of ICI and FI increases on roads with larger speed limit. As compared to the road with speed limit of 30 mph, pedestrians on road with speed limit over 65 mph are more likely to be involved in ICI (17.6% higher) and FI (7.7% higher). Nighttime was estimated to increase the likelihood of ICI (6.7% higher) and FI (2.9% higher) compared with daytime, while daylight decreases the likelihood of ICI (5.6% lower) and FI (2.4% lower) compared with other light conditions (dark not lighted and dark lighted). This result reflects the importance of the bright light condition for decreasing the risk of severe injury of pedestrians in crashes at mid-blocks. In a bright light condition, both the vehicle driver and the pedestrian could recognize the upcoming danger more quickly so that they could take some rapid measures to avoid severe injuries. With respect to the number of lanes, the results of the ordered logit model show that the direct effects of different number of lanes on pedestrian injury severity are not statistically significant (at *p*-value = 0.05 level), thus the direct impact of these three explanatory variables are 0%. However, by influencing the pre-crash behaviors significantly, the number of lanes have indirect effects on the injury severity. As shown in Table 5, roads with four lanes decrease the probability of ICI by 0.3% as compared to roads with one or two lanes.

## 6. Conclusions

To examine the inter-relationships between the pedestrian injury severity at mid-blocks and its explanatory variables, the path analysis method of SEM was applied in this study. The multinomial logit model for pedestrian pre-crash behaviors and the ordered logit model for pedestrian injury severity were estimated based on eight years of data on vehicle–pedestrian crashes from NASS-GES. There are 3653 records in the dataset, which contains five categories of information including pedestrian characteristics, vehicle characteristics, roadway features, environmental conditions, and crash attributes. According to the marginal effects in the multinomial and the ordered logit models, the direct, indirect, and total effects of various explanatory variables on the injury severity were calculated. The results show that the pedestrian’s age and gender, speed limit of roadway, number of lanes, light condition, and road surface condition have indirect effects on the pedestrian injury severity by influencing the pre-crash behaviors of pedestrians. Although most of the indirect effects are relatively small compared with the direct effects, the results indicate that the indirect influence through pre-crash behaviors should be taken into consideration for better understanding the associations between various contributing factors and the pedestrian injury severity at mid-blocks.

Besides, the results in this study also reveal some useful findings, which are summarized as follows: (1) Compared with pedestrians at the base age (<25), there is a much lower probability of darting or running into road for pedestrians with the ages 25–45, 45–65, and >65 (27.3%, 31.1%, and 27.1%, respectively). (2) As compared to automobiles, heavier vehicles including light trucks, buses, and heavy trucks, tend to increase the probability of more severe pedestrian injuries and lighter vehicles (motorcycles) result in a lower likelihood of ICI (4.8% lower) and FI (2.1% lower). (3) Compared with the front point of the vehicle as the first point of impact in the vehicle–pedestrian crash, both the left side and right side decrease the likelihood of ICI and FI of the pedestrian at mid-blocks. (4) Higher speed limits of roads tend to increase the likelihood of ICI and FI, and, specifically, pedestrians on roads with speed limit over 65 mph are more likely to be involved in ICI (17.6% higher) and FI (7.7% higher) compared with the speed limit of 30 mph.

In a recent study [16], the pedestrian fatality risk in vehicle–pedestrian crashes at mid-blocks in China was analyzed based on the data of Beijing, in which the pedestrian pre-crash behavior could not be considered due to the lack of relevant records in the dataset. If detailed records of vehicle–pedestrian crashes of cities in China were available in the near future, further studies could be conducted. This study provides reference for relevant studies based on the data from China.

In summary, this study provides some valuable information for better understanding the pedestrian injury severity in vehicle–pedestrian crashes at mid-blocks and is expected to be helpful to improve the pedestrian safety. However, the heterogeneity of the pre-crash behavior type was not considered in this study. More refined crash data could be used to analyze the inter-relationships between the pedestrian injury severity and its explanatory variables in future studies.

## Figures and Tables

**Table 1:** Distribution of potential contributing factors at different severity levels.

Variable		No injury/ Possible Injury (NIPI)		Non-Incapacitating Evident Injury (NIEI)		Incapacitating Injury (ICI)		Fatal Injury (FI)	
		Count	Ratio	Count	Ratio	Count	Ratio	Count	Ratio
**Pedestrian Characteristics**									
Age	<25	94	2.57%	1308	35.81%	598	16.37%	58	1.59%
	25–45	32	0.88%	346	9.47%	307	8.40%	84	2.30%
	45–65	23	0.63%	225	6.16%	231	6.32%	65	1.78%
	>65	8	0.22%	114	3.12%	102	2.79%	58	1.59%
Gender	Male	103	2.82%	1255	34.36%	860	23.54%	201	5.50%
	Female	54	1.48%	738	20.20%	378	10.35%	64	1.75%
**Vehicle Characteristics**									
Vehicle Type	Automobile	110	3.01%	1474	40.35%	899	24.61%	149	4.08%
	Light truck	31	0.85%	423	11.58%	263	7.20%	90	2.46%
	Bus	0	0.00%	5	0.14%	7	0.19%	1	0.03%
	Heavy truck	5	0.14%	19	0.52%	24	0.66%	17	0.47%
	Motorcycle	5	0.14%	21	0.57%	16	0.44%	3	0.08%
**Roadway Features**									
Speed Limit	<30 mph	78	2.14%	1062	29.07%	484	13.25%	44	1.20%
	30–55 mph	73	2.00%	873	23.90%	724	19.82%	199	5.45%
	>60 mph	3	0.08%	24	0.66%	20	0.55%	22	0.60%
Roadway Geometry	Curve	11	0.30%	58	1.59%	55	1.51%	21	0.57%
	Straight	146	4.00%	1935	52.97%	1183	32.38%	244	6.68%
Number of Lanes	One lane	25	0.68%	331	9.06%	123	3.37%	10	0.27%
	Two lanes	81	2.22%	941	25.76%	550	15.06%	98	2.68%
	Three lanes	23	0.63%	312	8.54%	224	6.13%	58	1.59%
	Four lanes	14	0.38%	235	6.43%	182	4.98%	56	1.53%
	Five or more lanes	12	0.33%	154	4.22%	153	4.19%	43	1.18%
**Environmental Conditions**									
Time of Day	Nighttime	30	0.82%	444	12.15%	433	11.85%	168	4.60%
	Peak time	59	1.62%	787	21.54%	427	11.69%	66	1.81%
Light Condition	Daylight	105	2.87%	1294	35.42%	609	16.67%	59	1.62%
	Dark not lighted	18	0.49%	179	4.90%	177	4.85%	100	2.74%
	Dark lighted	25	0.68%	424	11.61%	402	11.00%	101	2.76%
Surface Condition	Dry surface	131	3.59%	1735	47.50%	1058	28.96%	228	6.24%
	Wet surface	21	0.57%	207	5.67%	151	4.13%	29	0.79%
**Crash Attributes**									
First Point of Impact	Front	91	2.49%	1200	32.85%	838	22.94%	196	5.37%
	Right side	35	0.96%	366	10.02%	155	4.24%	8	0.22%
	Left side	17	0.47%	220	6.02%	103	2.82%	10	0.27%
Pre-crash Behavior	Darting or running into road	74	2.03%	1018	27.87%	511	13.99%	70	1.92%
	Improper crossing	46	1.26%	612	16.75%	486	13.30%	110	3.01%
	Activity in road	11	0.30%	144	3.94%	85	2.33%	30	0.82%
	Inattentive	4	0.11%	21	0.57%	8	0.22%	1	0.03%
	Other action	22	0.60%	198	5.42%	148	4.05%	54	1.48%

**Table 2:** Results of the multinomial logit model for pre-crash behavior of the pedestrian.

Pre-Crash Behaviors	Darting or Running into Road		Improper Crossing			Activity in Road		Inattentive	
	$\beta_{\left(1\right)}$	Marginal Effect	$\beta_{\left(2\right)}$	Marginal Effect		$\beta_{\left(3\right)}$	Marginal Effect	$\beta_{\left(4\right)}$	Marginal Effect
constant	1.853 ***		1.060 ***			−0.376		−2.354 ***	
**Age**									
25–45	−1.357 ***	−0.273	−0.100	0.145		0.418 **	0.064	−0.747	−0.001
45–65	−1.241 ***	−0.311	0.354 **	0.218		0.436 *	0.049	0.114	0.005
>65	−1.408 ***	−0.271	0.346 *	0.292		0.152	0.045	−14.955	−0.132
**Gender**									
Female	−0.274 **	−0.047	0.020	0.052		−0.453 **	−0.022	−0.103	0.000
**Speed Limit**									
30–55 mph	−0.317 **	−0.056	0.072	0.074		−0.660 **	−0.036	−0.282	−0.001
>60 mph	−0.632	−0.069	−0.481	−0.017		0.048	0.032	−0.008	0.004
**Number of Lanes**									
Three lanes	0.224	−0.005	0.415 **	0.058		−0.167	−0.028	0.460	0.002
Four lanes	0.382 **	−0.002	0.660 ***	0.089		−0.251	−0.044	0.406	0.000
Five or more lanes	0.615 ***	0.011	0.806 ***	0.073		0.244	−0.023	1.199 **	0.006
**Time of Day**									
Nighttime	−0.573 ***	−0.077	−0.420 **	−0.026		0.564 **	0.062	−0.199	0.002
**Light Condition**									
Daylight	−0.038	0.066	−0.473 ***	−0.075		−0.597 **	−0.024	0.437	0.006
**Surface Condition**									
Dry surface	0.393 **	0.071	0.046	−0.046		0.192	0.001	−0.226	−0.004
***Model Statistics***									
Log-likelihood			−3978.39						
LR Chi-square			884.06						
Prob > Chi-square			0.000						
Pseudo R2			0.100						

Note. The reference category for pre-crash behavior is “Other action”. * Significant at a 95% confidence level. ** Significant at a 99% confidence level. *** Significant at a 99.9% confidence level.

**Table 3:** Results of the ordered logit model for the pedestrian injury severity.

Variables	$\beta_{2}$	*p*-Value	Marginal Effect			
			NIPI	NIEI	ICI	FI
constant(1)	−3.152					
constant(2)	0.568					
constant(3)	3.004					
**Age**						
25–45	0.374 ***	<0.001	−0.015	−0.065	0.055	0.024
45–65	0.613 ***	<0.001	−0.025	−0.106	0.091	0.040
>65	1.030 ***	<0.001	−0.042	−0.178	0.152	0.067
**Gender**						
Female	−0.251 ***	<0.001	0.010	0.043	−0.037	−0.016
**Vehicle Type**						
Light truck	0.261 ***	0.001	−0.011	−0.045	0.039	0.017
Bus	1.001 **	0.049	−0.040	−0.173	0.148	0.065
Heavy truck	0.941 ***	<0.001	−0.038	−0.162	0.139	0.061
Motorcycle	−0.323	0.301	0.013	0.056	−0.048	−0.021
**Speed Limit**						
30–55 mph	0.455 ***	<0.001	−0.018	−0.078	0.067	0.030
>60 mph	1.190 ***	<0.001	−0.048	−0.205	0.176	0.077
**Number of Lanes**						
Three lanes	−0.095	0.363	0.004	0.016	−0.014	−0.006
Four lanes	0.140	0.192	−0.006	−0.024	0.021	0.009
Five or more lanes	0.238	0.051	−0.010	−0.041	0.035	0.015
**Time of Day**						
Nighttime	0.452 ***	<0.001	−0.018	−0.078	0.067	0.029
**Light Condition**						
Daylight	−0.407 ***	<0.001	0.016	0.070	−0.060	−0.026
**Surface Condition**						
Dry surface	0.202 *	0.039	−0.008	−0.035	0.030	0.013
**First Point of Impact**						
Right side	−0.637 ***	<0.001	0.026	0.110	−0.094	−0.041
Left side	−0.450 ***	<0.001	0.020	0.086	−0.074	−0.032
**Pre-crash Behavior**						
Darting or running into road	−0.266 *	0.013	0.010	0.048	−0.039	−0.019
Improper crossing	−0.230 *	0.044	0.008	0.042	−0.033	−0.016
Activity in road	−0.438 **	0.006	0.017	0.076	−0.065	−0.029
Inattentive	−0.930 *	0.015	0.046	0.143	−0.138	−0.051
***Model Statistics***						
Log-likelihood		−3471.56				
LR Chi-square		529.86				
Prob > Chi-square		0.000				
Pseudo R2		0.071				

Note. *, **, and *** denote significance at the 95%, 99%, and 99.9% confidence levels, respectively.

**Table 4:** Direct, indirect, and total effects of variables on the Incapacitating injury (ICI).

Independent Variables	Direct Effect	Effect on the Pre-Crash Behavior				Effect of Pre-Crash Behaviors on ICI				Indirect Effect on ICI				Total Effect
		$\lambda_{1}$	$\lambda_{2}$	$\lambda_{3}$	$\lambda_{4}$	$\varphi_{1}$	$\varphi_{2}$	$\varphi_{3}$	$\varphi_{4}$	$\lambda_{1}\varphi_{1}$	$\lambda_{2}\varphi_{2}$	$\lambda_{3}\varphi_{3}$	$\lambda_{4}\varphi_{4}$	
**Age**														
25–45	5.5%	−27.3%		6.4%		−3.9%	−3.3%	−6.5%	−13.8%	1.1%	0.0%	−0.4%	0.0%	6.1%
45–65	9.1%	−31.1%	21.8%	4.9%		−3.9%	−3.3%	−6.5%	−13.8%	1.2%	−0.7%	−0.3%	0.0%	9.3%
>65	15.2%	−27.1%	29.2%			−3.9%	−3.3%	−6.5%	−13.8%	1.1%	−1.0%	0.0%	0.0%	15.3%
**Gender**														
Female	−3.7%	−4.7%		−2.2%		−3.9%	−3.3%	−6.5%	−13.8%	0.2%	0.0%	0.1%	0.0%	−3.4%
**Speed Limit**														
30–55 mph	6.7%	−5.6%		−3.6%		−3.9%	−3.3%	−6.5%	−13.8%	0.2%	0.0%	0.2%	0.0%	7.2%
>60 mph	17.6%					−3.9%	−3.3%	−6.5%	−13.8%	0.0%	0.0%	0.0%	0.0%	17.6%
**Number of Lanes**														
Three lanes			5.8%			−3.9%	−3.3%	−6.5%	−13.8%	0.0%	−0.2%	0.0%	0.0%	−0.2%
Four lanes		−0.2%	8.9%			−3.9%	−3.3%	−6.5%	−13.8%	0.0%	−0.3%	0.0%	0.0%	−0.3%
Five or more lanes		1.1%	7.3%		0.6%	−3.9%	−3.3%	−6.5%	−13.8%	0.0%	−0.2%	0.0%	−0.1%	−0.4%
**Time of Day**														
Nighttime	6.7%	−7.7%	−2.6%	6.2%		−3.9%	−3.3%	−6.5%	−13.8%	0.3%	0.1%	−0.4%	0.0%	6.7%
**Light Condition**														
Daylight	−6.0%		−7.5%	−2.4%		−3.9%	−3.3%	−6.5%	−13.8%	0.0%	0.2%	0.2%	0.0%	−5.6%
**Surface Condition**														
Dry surface	3.0%	7.1%				−3.9%	−3.3%	−6.5%	−13.8%	−0.3%	0.0%	0.0%	0.0%	2.7%

Note. For $\lambda_{i}$ and $\varphi_{i}$: *i* = 1, Darting or running into road; *i* = 2, Improper crossing; *i* = 3, Activity in road; *i* = 4, Inattentive.

**Table 5:** Direct, indirect, and total effects of explanatory variables on the pedestrian injury severity.

Independent Variables	No Injury/Possible Injury (NIPI)			Non-Incapacitating Evident Injury (NIEI)			Incapacitating Injury (ICI)			Fatal Injury (FI)		
	Direct	Indirect	Total	Direct	Indirect	Total	Direct	Indirect	Total	Direct	Indirect	Total
**Age**												
25–45	−1.5%	−0.2%	−1.7%	−6.5%	−0.8%	−7.3%	5.5%	0.6%	6.1%	2.4%	0.3%	2.7%
45–65	−2.5%	−0.1%	−2.6%	−10.6%	−0.2%	−10.8%	9.1%	0.2%	9.3%	4.0%	0.1%	4.1%
>65	−4.2%	0.0%	−4.2%	−17.8%	−0.1%	−17.9%	15.2%	0.1%	15.3%	6.7%	0.0%	6.7%
**Gender**												
Female	1.0%	−0.1%	0.9%	4.3%	−0.4%	3.9%	−3.7%	0.3%	−3.4%	−1.6%	0.2%	−1.4%
**Speed Limit**												
30−55 mph	−1.8%	−0.1%	−1.9%	−7.8%	−0.5%	−8.3%	6.7%	0.5%	7.2%	3.0%	0.2%	3.2%
>60 mph	−4.8%	0.0%	−4.8%	−20.5%	0.0%	−20.5%	17.6%	0.0%	17.6%	7.7%	0.0%	7.7%
**Number of Lanes**												
Three lanes		0.0%	0.0%		0.2%	0.2%		−0.2%	−0.2%		−0.1%	−0.1%
Four lanes		0.1%	0.1%		0.4%	0.4%		−0.3%	−0.3%		−0.1%	−0.1%
Five or more lanes		0.1%	0.1%		0.4%	0.4%		−0.4%	−0.4%		−0.2%	−0.2%
**Time of Day**												
Nighttime	−1.8%	0.0%	−1.8%	−7.8%	0.0%	−7.8%	6.7%	0.0%	6.7%	2.9%	0.0%	2.9%
**Light Condition**												
Daylight	1.6%	−0.1%	1.5%	7.0%	−0.5%	6.5%	−6.0%	0.4%	−5.6%	−2.6%	0.2%	−2.4%
**Surface Condition**												
Dry surface	−0.8%	0.1%	−0.7%	−3.5%	0.3%	−3.2%	3.0%	−0.3%	2.7%	1.3%	−0.1%	1.2%
